# Internal Hemorrhage*Read at annual meeting of West Texas Medical Society at San Antonio, Texas.

**Published:** 1898-11

**Authors:** F. Paschal

**Affiliations:** San Antonio, Texas


For Texas Medical Journal.

### Internal Hemorrhage.*

BY F. PASCHAL, M. D., SAN ANTONIO, TEXAS.

The physician or surgeon has little to fear in a case of external hemorrhage, provided he gets to the patient in time. because usually some measure can be employed to arrest it. When the hemorrhage is internal it is different, for then we have to deal with conditions that are entirely changed, and instead of seeing we have to judge by symptoms and signs of what is going on within the hidden recesses, and we are never certain of the source of the bleeding, and even the symptoms and signs are by no means pathognom-

*Read at annual meeting of West Texas Medical Society at San Antonio, Texas.

onic of hemorrhage. As the surgical treatment of internal hemorrhage will only be undertaken in extreme cases, I may be pardoned for referring to the way that nature arrests bleeding, for I do not believe any of us can be too familiar with the process, and the better we understand it the less will be the danger of making mistakes.

The means that nature adopts to stop bleeding are, in the first instance, temporary, but afterwards become permanent. If the vessel be small they are sufficient to stay the hemorrhage without the surgeon's interferring, and whatever the size of the vessel, his operations are materially assisted by the effort nature makes, though it may be an ineffectual one, to prevent a fatal escape of blood.

In the first place, there is a coagulation of, and alteration in the constitution of the blood.

Second, a diminution in the heart's action, and consequently of the pressure on the inner coat of the vessel.

Third, certain changes effected in and around the vessel.

The coagulation of the blood in and around the wounded artery is the first and most important means adopted to arrest hemorrhage.

The blood that escapes from a wounded artery has, from the first, a tendency to glaze or coagulate about the cut vessel, so as to offer a mechanical obstacle to the further escape of the fluid. This, of itself, is sufficient in smaller vessels to arrest the hemorrhage, the more so as the last flowing blood is more coagulable than the first.

The diminution in the force of the heart's action, owing to the patient becoming faint or collapsed, exercises a very material influence in arresting the flow of blood. Each lowered systole of the heart increases the tendency to the formation of a coagulum in the cut end of the vessel, and diminishes the chances of its being washed away. The collapse consequent upon the loss of blood should be looked on as one of nature's methods of arresting hemorrhage, and should, therefore, not be too hastily counteracted by stimulants, or in any other way.

The final arrest of bleeding is dependent upon changes which take place in and around the vessel itself.. An artery when cut across, immediately retracts within its sheath, the interior of which is rough and uneven. As the blood flows over the roughened surface of the sheath, it becomes entangled upon its fibers, and tends to coagulate upon them. This coagulum projects beyond the cut end and forms the external coagulum. By the artery contracting, the calibre of the vessel is diminished, and in proportion as the vessel is obstructed by the external clot the greater the difficulty with which the blood is propelled through it until it ceases to escape, and a deposit of fibrin takes place, which plays an important part in the permanent arrest of hemorrhage. Finally, inflammatory changes take place in the coats of the vessel and adheres their surfaces together and permanently closes it.

The constitutional effects of the loss of blood depend upon the amount lost and upon individual susceptibility; for one person will bear the loss of blood badly, while another might lose a greater amount without suffering serious consequences. After severe bleeding, the quantity of blood is quickly regained, but it is doubtful whether the quality of this complex fluid is ever restored, and it always leaves its impress of anemia upon the persons who have lost it, and predisposes them to many forms of disease.

The symptoms of hemorrhage depend upon the amount of blood lost. When it is large, the face becomes pinched and palid, the conjunctivæ, lips and ears blanched, the integument shriveled, the skin bathed in a cold, clammy sweat. From anemia of the brain, humming or roaring sounds are heard, the pupils are dilated, a thick mist or even darkness, alternating with flashes of light, comes before the eyes. General sensibility is diminished, and even unconsciousness, with syncope or convulsions, follow. The faint whispering voice, the feeble sighing respiration, the marked dysponea and restlessness, and the small, frequent flutterings, almost imperceptible pulse, make a picture too vivid to ever be forgotten, and those of us who have watched a death from hemorrhage have felt like saying—

> "Dust to its narrow house beneath;
> Soul to its place on high!
> They who have seen thy face in death
> No more may fear to die."

But however clearly these symptoms point to internal hemorrhage, they can all be simulated by profound shock and collapse from internal injury. In arriving at a diagnosis a quick and careful study of the case must be made, for a nice point in judgment may confront us, for if the symptoms are due to collapse from shock, any surgical interference would hasten death, whereas, if withheld might save life; but on the other hand, if the symptoms are due to hemorrhage, any beat of the heart may send the last drop of blood that can be spared through the bleeding vessel. Here, as in every emergency case, the surgeon who dares to do his duty is the one most likely to do what is right. There is no time in these cases for lengthy consultations—the surgeon, if he would save life, must act, and act quickly.

As the thoracic and abdominal cavities are those in which hemorrhage most frequently occur, I shall confine my remarks to them. The most frequent cause of hemorrhage in the thoracic cavity is from wound of an intercostal artery, wound of the lung tissue, wound of the internal mammary artery, and wound of the heart. When internal hemorrhage follows a wound of the chest, it is not always easy to say whether the bleeding is from a wounded intercostal artery or from the lung tissue. If the external wound is large enough to enable the bright red blood to be seen escaping in spurts, synchronous with the action of the heart, it can be differentiated from the oozing of dark blood from the lung. But the external wound may be small, and the intercostal artery or lung tissue may bleed almost entirely within the cavity, and our chief reliance for a diagnosis must be placed on signs and symptoms. If the bleeding is from the lung tissue, there is usually cough, with expectoration of frothy blood, and unless the wound involves both intercostal artery and lung, there will be no expectoration of blood if the intercostal artery is alone injured. The physical signs are of the utmost importance to enable us to differentiate hemorrhage from shock and collapse. The signs correspond to the accumulation of other fluids within the chest. When blood is present to any considerable extent, there will be dullness in the most pendent portions of the chest, absence of vocal fremitus, absent or diminished respiratory murmur, with reappearance of normal physical signs, when the fluid is made to gravitate by posture.

The diagnosis of the probable source of the hemorrhage having been made, the question is, what shall be done? Assuming that the routine plan of treatment by rest, external application of cold, opium, and by drugs of doubtful efficacy, such as ergot, acetate of lead, gallic acid, and all such truck, have been employed and the hemorrhage still continues, and if the bleeding is from an intercostal artery, I think the most rational plan would be to resect a part of the rib near the wound, secure the artery, incise the pleura, let out the blood, wash out the cavity with warm salt solution, and if need be, drain. To try to plug a wound or to enlarge it and secure the artery and still leave the chest half-full of blood, with all of its attendant dangers, does not appeal to me as good surgery, and if any enlarging is to be done, I believe it would be better to do it by resecting the rib, as by so doing the situation is mastered.

If the hemorrhage is from the lung tissue, what should be done? Assuming, as before, that the usual means have been employed and have failed, I think the plan would be to resect a part of two or more ribs, catch the lung with volsellum forceps, draw the lung as near to the surface as possible, and if the wound in the lung is large enough to admit of its being plugged, plug it with aseptic gauze, and if the wound is not large enough, enlarge it by incision or dilatation and plug it, bringing the end of the gauze out of the chest. I can see no reason why this method of treatment of wounds of the lung should not be employed, even in those cases where the hemorrhage is manifested by profuse hemoptysis. Indeed, I can see no good reason why the surgeon should, if necessary, hesitate to follow the route the foreign body has traversed, for the damage has been done, and he should try to compress the cut vessel at all hazards. In all probability the wound was made with the full intention to cause death, and if our patient dies while we stand idly by waiting and hoping, we may be after all the guilty party. If the internal mammary artery is the cause of hemorrhage, it should be ligated, and in order to do this several of the cartilages may have to be divided. Hemorrhage from this artery may take place in the mediastinum or pleural cavity.

Hemorrhage into the pericordial sac may be due to wounds of the heart, which we all know are not necessarily fatal. When the accumulation is sufficient to seriously enbarrass the action of the heart, I think the sac should be incised, and it might be necessary to pass a needle into the heart muscle and sew the wound; however, one is not very apt to be called upon to do this, for either the wound will be insignificant or it will be fatal.

The surgeon is sometimes responsible for wounds of an intercostal artery. The due attention he will pay to the location of this artery when he performs paracentesis, will be the only preventive treatment of internal hemorrhage into the chest that he can employ.

Hemorrhage into the abdominal cavity may be traumatic, idiopathic, or of part operative origin. There may be an external wound in the abdomen to indicate the possible existence of internal bleeding, or there may be no external wound, the hemorrhage being caused by rupture of an organ within the abdomen, caused by a blow; or hemorrhage may occur from an aneurism, from a ruptured ectopic pregnancy, or follow surgical operations within the abdomen. However the hemorrhage may be caused, its diagnosis is by no means always easy. As stated before, the symptoms following shock and collapse may simulate those of hemorrhage so closely as to render it difficult to arrive at a correct conclusion. There are certainly cases that test to the fullest extent the ability to make them out and properly treat them.

The treatment of hemorrhage into the abdominal cavity will depend upon the amount of the hemorrhage and upon its cause. Where the amount of bleeding is small, it can safely be left to nature, as the blood will be absorbed. If, however, there has been a penetrating wound of the abdomen, we have no assurance that the intestines or some blood vessel or organ has not been wounded, and possibly the best plan would be to enlarge the wound, or do a laparotomy, and if possible assure ourselves of the condition within the cavity, and if hemorrhage is found to be going on, its origin should be ascertained and arrested. The surgeon who withholds from opening the abdomen when there are indications for doing so, or the one who delays an intra-abdominal operation when even a little delay may put the patient beyond hope, or seriously jeopardize the chances of recovery, assumes a responsibility which is certainly very great, and if he realizes that too much time was lost, he will, if he does not care to keep on blundering, profit by his mistake, as we should all do if we have sense enough to know when we make them. It is especially when hemorrhage is going on within the abdomen that these remarks should hold good, for these of all cases that we are called upon to treat will brook no delay. The prevention of hemorrhage within the abdomen sometimes depends upon ourselves; for instance, in cases of ectopic pregnancy. If we are consulted before rupture of the sac has taken place, we should, in the majority of cases, be able to recognize the existence of ectopic pregnancy and urge an operation before the sac is allowed to rupture. Some of the causes of hemorrhage after abdominal operations are slipping of a ligature in tying off omentum, or mesentery or ovaries and tubes. Another cause of hemorrhage is tearing of the broad ligament after removing the tube and ovary. The usual practice in removing tubes and ovaries is to tie off the tube and ovary together. In doing this a fold of the broad ligament is included in the stump, and after transfixing the mass in half and tying, it is divided, and some tie each vessel in the stump to doubly insure against hemorrhage in case the ligature around the stump should slip. Ordinarily this way of dealing with the tube and ovary gives no trouble, but notwithstanding the most careful tying I have seen frightful and fatal hemorrhage afterward. This not being due to any faulty tying, but to a tearing of the broad ligament. The manner in which this occurs is possibly as follows: The broad ligament having been put on the stretch on account of the fold taken up with the tube and ovary, it begins to tear near the stump, and a rent is made which pulls away from the stump and carries with it numerous vessels, which pour out an enormous quantity of blood, and which may be quickly fatal. In order to avoid the danger of the broad ligament tearing, it might be better, when it is possible to do it, to tie the tube and ovary off separately, especially the tube from the broad ligament, just as we tie off the appendix from its mesenteric attachment. Of course, it will not be possible to do this in all cases.

But however careful one may be in doing operations within the abdomen, hemorrhage is liable to occur, and when it does occur and can be made out, the abdomen should be reopened and an effort made to arrest bleeding.

There is an important point to be remembered in internal abdominal hemorrhage; it is that however quickly we make our preparations to open the abdomen, it consumes time, and during our preparations the bleeding is, of course, going on. We should remember the rule here that holds good in external hemorrhage—put the fingers on the bleeding point or main artery—and we should follow this rule and compress the abdominal aorta, and have an assistant keep it compressed until the abdomen is opened, and after it is opened an assistant should compress it until we are assured that the bleeding point has been found and is stopped.

Some of you gentlemen present will, no doubt, say we know all of this; well, no doubt you do, and I thought that I knew it until put to the test, and I then found that if I did know it I did not put it into practice, and deeply regret not having done so, and if my mistake in not having compressed the abdominal aorta can make you remember to compress it should you ever be so unfortunate as to meet with a severe intra-abdominal hemorrhage, I shall feel amply repaid for having imposed on your good natures in asking you to listen to this paper.

